# Recent Advances in In Vivo Neurochemical Monitoring

**DOI:** 10.3390/mi12020208

**Published:** 2021-02-18

**Authors:** Chao Tan, Elaine M. Robbins, Bingchen Wu, Xinyan Tracy Cui

**Affiliations:** 1 Department of Bioengineering, University of Pittsburgh, Pittsburgh, PA 15261, USA; cht117@pitt.edu (C.T.); emr72@pitt.edu (E.M.R.); biw15@pitt.edu (B.W.); 2 Department of Chemistry, University of Pittsburgh, Pittsburgh, PA 15260, USA; 3 Center for Neural Basis of Cognition, Pittsburgh, PA 15213, USA; 4 McGowan Institute for Regenerative Medicine, Pittsburgh, PA 15219, USA

**Keywords:** neurotransmitters, biosensors, neurochemical sensor, analytical neurochemistry, in vivo, brain

## Abstract

The brain is a complex network that accounts for only 5% of human mass but consumes 20% of our energy. Uncovering the mysteries of the brain’s functions in motion, memory, learning, behavior, and mental health remains a hot but challenging topic. Neurochemicals in the brain, such as neurotransmitters, neuromodulators, gliotransmitters, hormones, and metabolism substrates and products, play vital roles in mediating and modulating normal brain function, and their abnormal release or imbalanced concentrations can cause various diseases, such as epilepsy, Alzheimer’s disease, and Parkinson’s disease. A wide range of techniques have been used to probe the concentrations of neurochemicals under normal, stimulated, diseased, and drug-induced conditions in order to understand the neurochemistry of drug mechanisms and develop diagnostic tools or therapies. Recent advancements in detection methods, device fabrication, and new materials have resulted in the development of neurochemical sensors with improved performance. However, direct in vivo measurements require a robust sensor that is highly sensitive and selective with minimal fouling and reduced inflammatory foreign body responses. Here, we review recent advances in neurochemical sensor development for in vivo studies, with a focus on electrochemical and optical probes. Other alternative methods are also compared. We discuss in detail the in vivo challenges for these methods and provide an outlook for future directions.

## 1. Introduction

### 1.1. Neurochemicals

The brain is the most complex organ in the human body, consisting of tens of billions of neurons and hundreds of trillions of synaptic connections. The brain is responsible for many functions, including movement, sensation, awareness, and memory. Diseases of the nervous system are a great threat to public health. One in six people is suffering from a neurological disease and one third of the population will be affected by a brain disorder at some point in their lifetime. Most neurological disorders are associated with abnormal activity or imbalances of neurochemicals. Neurochemicals are chemical substances that participate in brain activity. There are many categories of neurochemicals, including neurotransmitters and gliotransmitters that allow for fast cell-to-cell chemical communication, hormones and cytokines that are involved in slower timescale communication, neuropeptides, metabolism substrates and metabolites, and salts that facilitate cell firing. A complex web of hundreds of neurochemicals is required for the normal functioning of the central nervous system. For example, the catecholamine neurotransmitter dopamine (DA) operates at several different timescales and plays an essential role in behavioral and cognitive functions, such as movement, motivation, attention, and learning [1,2,3,4,5]. However, pathological changes in the concentrations of neurochemicals such as DA can result in a variety of symptoms, such as the loss of motor control and cognitive deficits shown in Parkinson’s disease patients [6,7,8]. To better understand the role of DA in mediating and modulating neural transmission and consequently affecting brain function, many DA sensors have been developed in order to track DA concentrations in different regions of the brain. For example, a microarray electrode that integrates a DA chemical sensor with electrophysiological recording channels has helped illustrate the mechanism by which deep brain stimulation (DBS) alleviates Parkinson’s disease symptoms by modulating the release of DA [9].

Beside neurotransmitters, many other neurochemicals are also involved in normal communication, organization, and function in the brain. For instance, ascorbic acid (AA) is a powerful antioxidant that can scavenge free radicals in the brain and reduces the likelihood of tissue damage or disease resulting from oxidative free radicals [10]. AA is of interest as a potential treatment for oxidative stress-related damage following traumatic brain injury (TBI), through its potential to react with radical oxygen and nitrogen species. Monitoring AA concentrations in the brain is a critical step in the development of AA-related treatments for oxidative stress [11]. Additionally, gliotransmission has recently become a hot area of research in neuroscience. There is evidence that astrocytes can chemically communicate bidirectionally with other cells using glutamate (Glu), D-serine, lactate, and adenosine triphosphate (ATP) [12,13,14,15]. The lactate shuttle hypothesis—the idea that astrocytes deliver lactate to energy-stressed neurons—is of particular interest in TBI research. Neurons in an injured brain that are starved of glucose may switch to a lactate-based metabolism, relying on local astrocytes for their energy needs [16,17]. Lactate biosensors have been used to verify that hypothesis and show that anesthetic agents can impair the brain’s lactate shuttle by interfering with astrocytic gliosis, and thus reduce the energy supply to neurons [18].

Despite the fact that trillions of dollars are spent annually to treat brain diseases such as Alzheimer’s disease, TBI, and epilepsy, there are still vital gaps in knowledge: there is no cure for many brain disorders, including Alzheimer’s, Parkinson’s, and Huntington’s disease. In fact, a diagnosis of Alzheimer’s disease can only be confirmed by a post-mortem examination. Additionally, even though TBI is the primary cause of death for people under 45 years of age, there is currently no pharmaceutical treatment available. To develop more accurate diagnosis of neurological disorders and better treatments, the advancement of existing neurochemical sensors and the development of new ones are absolutely essential aims.

### 1.2. In Vivo Neurochemical Sensing Challenges

Sensors are valuable tools in biomedical research that provide quantitative monitoring of real time chemical changes in vivo and in some cases clinically [19]. A biosensor consists of at least two functional components: a molecular recognition element that selectively interacts with the target analyte and a transducer that converts the bio-recognition information into a measurable electrical, optical, or thermal signal. To evaluate the performance of a biosensor, several metrics are usually taken into consideration, including sensitivity, limit of detection (LOD), selectivity, rise time, long-term stability, and biocompatibility. Sensitivity is the signal amplitude to analyte concentration ratio, and high sensitivity leads to a low limit of detection. Selectivity describes how well a biosensor detects an analyte when coexisting with interferents. Rise time is defined in many electrochemical studies as the time required to rise from 10% to 90% of the total signal amplitude (T_10–90_). 

The design of a neurochemical sensor is difficult because an ideal neurochemical sensor must be able to distinguish its target from interference in the complex neurological environment, respond on an appropriate timescale, and exhibit long-term stability and biocompatibility. Neurochemical sensors designed for in vivo use have several concerns that must be addressed that may not matter for other applications. The most obvious concern is size; the sensor must be small enough to be insertable into tissue while causing minimal damage. However, mechanical robustness is also important for the sensor to be able to penetrate the brain without breaking or bending, especially when applied to deep brain measurements or long-term measurements in animals. Additionally, the neural environment is highly complex, with thousands of potentially interfering compounds; therefore, it is imperative to be confident in the selectivity of the sensor. Timescales of neurochemical events are highly variable and must also be kept in mind. Monitoring DA transients requires sub-second timescales, whereas some applications need only minute or hour timescales [20,21]. In cases where long-term monitoring is desired especially, robustness in response to fouling and inflammatory processes is necessary for a sensor to survive in vivo.

#### 1.2.1. Quantitative Analysis

Quantitative analysis is the monitoring of a target analyte’s dynamics at either basal levels or in stimulated conditions. Analytical tools used in vivo must exhibit high sensitivity, selectivity, and spatiotemporal resolution because in vivo neurochemical sensing involves low concentrations in a complex and harsh local environment, meaning sensor design choices must be carefully considered. Sensitivity loss and selective membrane degradation after implantation usually have the most effect on the quantitative results of neurochemicals. Many neurochemicals of interest are present in the brain at pM levels or less and can have huge dynamic changes because of stimuli, behaviors, or disease states. For example, basal levels of DA in the brain range from 1–200 nM and can increase to several µM in a L-DOPA treated Parkinson’s patient or during stimulated release events [22,23,24,25]. Therefore, an ideal neurochemical sensor must be endowed with a low limit of detection and a wide measurement range. Additionally, the brain environment is extremely complex with potentially thousands of interfering compounds; selectivity must be a priority when designing a neurochemical sensor and choosing a detection technique. Timescales of measurements are also highly dynamic and require a large range of temporal resolution for different events. For example, neuron firing events require very fast-responding sensors; dopaminergic neurons can fire in a bursting pattern at upwards of 100 Hz, resulting in detectable sub-second DA transients [26,27,28]. On the other hand, a basal-level DA fluctuation, also known as a tonic release, occurs at the time scale of seconds to minutes and serves to modulate the firing pattern [29]. Meanwhile, disease model monitoring may necessitate a sensor robust enough to survive days or weeks of implantation and remain responsive. We discuss below the advantages and disadvantages of several detection methods and sensor types in terms of sensitivity, selectivity, and timescale.

#### 1.2.2. The Inflammatory Response

Inflammatory responses are one of the main challenges with in vivo chemical sensing. Inflammatory responses occur due to the brain’s natural defensive mechanism to a foreign body. Foreign body responses in the brain involve a cascade of events, such as activation of microglial cells that encapsulate the implanted surface, and activation and infiltration of inflammatory cells, including neutrophils, monocytes, and macrophages. Implant surfaces are also susceptible to non-specific adsorption of proteins which can not only directly affect the sensor’s sensitivity, but also trigger a cascade of inflammatory responses, as some of these plasma proteins are neurotoxic or pro-inflammatory. A detailed discussion on insertion-injury-induced, molecular-level tissue responses and acute or chronic inflammation cascades can be found in our previous review [30]. Additionally, the inflammatory cells may release reactive oxygen species (ROS), which may not only damage the surrounding tissue but also degrade metallic or carbon sensors and the insulations [31,32,33]. Furthermore, cytokines released as a result of the inflammatory response have significant effects on the metabolism of neurotransmitters such as serotonin (5-HT), DA, and Glu by affecting their synthesis, release, and uptake [34]. Considering that the inflammatory response not only affects the sensor response, but also causes fluctuation of neurochemical concentrations in the vicinity of sensors, it would be advantageous to minimize the inflammatory response. 

In this review, we will give an overview of various types of sensors employed for in vivo neurochemical sensing, including electrochemical sensors, optical sensors, and a few alternative neurochemical sensing approaches. For each sensor category, we briefly compare the different detection mechanisms and review the recent advances in material development and device fabrication. We also discuss the additional difficulties of in vivo applications and present a detailed outlook on the future of in vivo neurochemical sensors.

## 2. Electrochemical Sensors

Electrochemical sensors were some of the first sensors developed for neurochemicals and are still widely employed. Electrochemical detection methods have the advantage of high spatial and temporal resolution, and depending on the type of electrode used, may be quick and inexpensive to manufacture. However, typically only electroactive neurochemicals can be monitored with electrochemistry, although we will also discuss modified electrodes that can be employed to detect non-electroactive compounds. Different electrochemical techniques have been used to characterize microsensors and detect analytes of interest, which include but are not limited to amperometry, fast-scan cyclic voltammetry, differential pulse voltammetry, and square wave voltammetry. In this section, we focus on a review of these four methods as they have been well accepted and extensively used in vivo and in vitro.

### 2.1. Electrochemical Detection Methods

#### 2.1.1. Chronoamperometry

**Figure 1:**
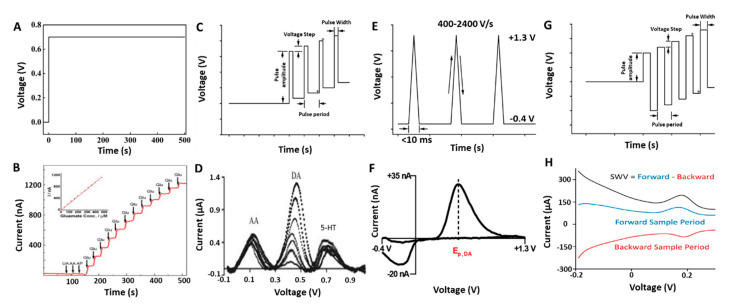
(**A**) Waveform of CA at a biased voltage of +0.7 V. (**B**) Current response of a Pt/PPy/MWCNT/glutamate oxidase electrode to 250 µM acetaminophen (AP), UA, AA, and successive injections of 20 µM Glu. Adapted with permission from [63]. (**C**) Waveform of DPV, indicating the current measurement periods. (**D**) Baseline-corrected DPV of a mixture of 1 mM AA, 10 µM 5-HT, and 0.1–10 µM DA from a carbon nanofiber electrode. Adapted with permission from [44]. (**E**) Waveform of FSCV between −0.4 and +1.3 V. (**F**) A background-subtracted DA signal from a CFM. Adapted with permission from [64]. (**G**) Waveform of SWV, indicating the current measurement periods. (**H**) SWV measurement of a 1 μM DA (black), forward (blue) and reverse (red) current responses. Adapted with permission from [25].

Chronoamperometry (CA) monitors the gain or loss of electrons in the presence of a fixed potential, with current (A) as the y axis and time (sec) as the x axis; see Figure 1A. CA can be performed using either a three-electrode set up consisting of a working electrode, a reference electrode, and a counter electrode, or a two-electrode set up where the reference and counter are shorted together. The working electrode is often a carbon-based material, but in many studies can also be metal-based—e.g., platinum or gold, due to their advantages in conductivity [35], biocompatibility [36], stable potential window [37], and electrocatalytic activity [38,39,40]. The reference electrode provides a stable standard potential so that the voltage at the working electrode can be controlled. The counter electrode is usually made of an inert metal such as platinum to prevent excessive interaction with the electrolyte solution or tissue environment, though in many in vivo applications, a stainless-steel bone screw anchored in the skull is utilized instead. In CA, a pre-stabilization time (>10 min) is usually necessary to obtain a steady baseline, after which electroactive analytes or stimulations can be introduced; see Figure 1B. 

CA has an obvious advantage over other electrochemical methods in terms of temporal resolution and recording time. The temporal resolution of CA makes it possible to study neurochemical events on millisecond timescales. The simple mechanism of CA grants it the ability to continuously record for several hours [41,42].

The main disadvantage of CA methods is the lack of selectivity; multiple electroactive species may participate in redox reactions on the electrode surface, making it impossible to determine how much current is actually coming from the reaction of interest. Some methods have been developed to improve measurement selectivity. In CA, the selectivity is somewhat tunable, since the applied potential can be changed to a value that is more sensitive to the analyte of interest but not to interferents [43]. Additionally, the application of coatings that are catalytic for the target redox reaction can help push the required detection potential away from that of potential confounding reactions. Other methods such as the self-referencing or coating of a permselective membrane are also commonly used in in vivo studies, as detailed in Table 1.

#### 2.1.2. Differential Pulse Voltammetry

Differential pulse voltammetry (DPV) waveforms consist of a series of constant-amplitude pulses superimposed on a slow-changing base potential waveform. The determined current is the difference between two points: before the application of a pulse and at the end of a pulse phase; see Figure 1C. DPV is a highly sensitive analytical tool that allows for direct detection of basal analytes at low concentrations, because the Faradaic to charging current ratio is high. The short pulse time increases the measured Faradaic current, and the differential method minimizes the background charging current.

DPV has been used as a tool to improve selectivity by separating the oxidation peaks when measuring a mix of analyte and interferents in one cell. For example, a nitric acid-treated, vertically aligned carbon nanofiber electrode was used for simultaneous detection of DA, 5-HT, and AA [44]. In contrast to CA, where the oxidation potentials of AA, DA, and 5-HT may overlap, DPV with carbon nanofiber electrodes was able to distinguish three distinct peaks at 0.13, 0.45, and 0.7 V vs. Ag/AgCl; see Figure 1D. In addition, Zhu et al. performed an in vivo study on the striata of anesthetized mice by modifying a carbon fiber microelectrode (CFM) with graphene-iron-tetrasulfophthalocyanine for enhanced electro-catalytic sensing of DA and 5-HT. In their study, DPV was able to distinguish the oxidation peaks of DA and 5-HT at 0.15 and 0.36 V, respectively [45]. Similar studies, both in vitro and in vivo, using microelectrodes and DPV to selectively detect neurochemicals have been reported [46,47,48,49].

#### 2.1.3. Square Wave Voltammetry

Square wave voltammetry (SWV) is a special type of DPV in which the waveform is a differential pulse superimposed on a staircase potential. The current collection point happens at the end of each peak and at the following valley of the square pulse. The decay of the capacitive current is much faster than Faradic current. As a result, SWV minimizes the interference from capacitive current. SWV also integrates both the oxidation and reduction peaks into a single peak readout which also improves the signal to noise ratio; see Figure 1G,H. SWV is advantageous in speed compared to DPV and capable of direct detection of basal levels of analytes. A detailed comparison between DPV and SWV can be found elsewhere [50].

In one study, a modified version of SWV developed by Oh et al., multiple cyclic square wave voltammetry (M-CSWV), was used and the sensor was fabricated by coating poly(3,4-ethylenedioxythiophene) (PEDOT): Nafion on CFM [51]. The reported LOD of the sensor is 0.17 nM with a temporal resolution of 10 s. They demonstrated successful detection of tonic DA in the rat striatum, reporting a concentration of 120 ± 8 nM.

#### 2.1.4. Fast Scan Cyclic Voltammetry

Fast scan cyclic voltammetry (FSCV) is cyclic voltammetry with a very high scan rate. The standard voltage-time waveform is a symmetrical triangle potential sweep; see Figure 1E. FSCV has been very successful in detecting electrochemically active species released in the brain. Compounds of interest are rapidly oxidized and reduced at the electrode/analyte solution interface and the electron transfer during the redox reaction induces a current change picked up by the instrument. FSCV provides high temporal resolution and good selectivity as a result of its ability to distinguish analytes based on the unique redox potentials of analytes, without the need to further coat electrodes with screening layers. FSCV is well accepted in the determination of neurochemicals, especially in the region where specific neurons account for the majority [52]. Due to the fast scan rate, FSCV intrinsically faces the problem of capacitive charging current. While Faradaic current is proportional to the square root of the scan rate, the non-Faradaic charging current changes linearly with the scan rate; therefore, the faster the scan rate, the lower the Faradaic/non-Faradaic current ratio. In order to separate the current from the reaction of interest from the capacitive current, a sample of the background must be taken and subtracted out. As a result, FSCV is a differential method that can only detect phasic responses. 

FSCV is one of the most used and mature electrochemical detection methods utilized in neurochemical sensing. Among the vast variety of neurochemicals detectible with FSCV, DA is the most well studied; see Figure 1F [24,53,54]. For more in-depth background, please see the referenced book chapter that explains in detail the procedures to use for FSCV detection of DA in different brain regions [55]. CFMs are the most commonly used electrodes for FSCV applications. Over the years, efforts have been made to change the waveform, scan rate, and electrode materials with the goal of improving FSCV’s sensitivity for different neurochemicals [56]. Sensitivity and signal to noise ratio improvements in DA detection were reported by increasing the scan rate from 400 V/s to 2400 V/s [57]. Jackson and co-workers applied FSCV to detect 5-HT in vivo. They compared the performance of bare CFM against Nafion-coated CFM and found that a Nafion-coated CFM was 20 times more sensitive to 5-HT than to DA. The reported 5-HT sensitivity was 5 nAµM^−1^_,_ compared to a typical bare CFM sensitivity of 1 nAµM^−1^ [58]. Additional FSCV waveforms to selectively detect histamine, adenosine, melatonin, hydrogen peroxide and others have been developed [59,60,61,62].

### 2.2. Materials for Electrochemical Sensors

#### 2.2.1. Carbon Based Electrodes

Carbon has been used as an electrode material in electrochemistry due to its unique properties, such as high electrical conductivity, chemical stability, easy processability, and low cost [65]. More recently, various carbon nanomaterials, such as carbon nanotubes (CNTs), graphene, and nanodiamond (ND) [66,67,68], have emerged as advanced electrochemical sensing platforms for the detection of neurochemicals in vivo. Carbon nanomaterials offer two key advantages: (1) increased surface area for reaction or support as a matrix for enhanced immobilization; (2) fast charge transfer and electrocatalytic behavior for neurochemical detection. 

**Figure 2:**
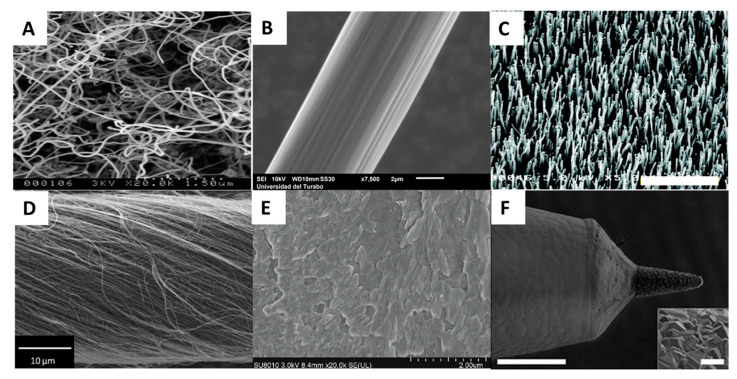
SEM pictures of (**A**) a randomly oriented carbon nanotube; the scale bar is 1.5 µm. Adapted with permission from [91]. (**B**) Exposed area of carbon fiber microelectrode; the scale bar is 2 µm. Adapted with permission from [92]. (**C**) As-grown vertically aligned carbon nanofibers; the scale bar is 6 µm. Adapted with permission from [93]. (**D**) 20° twisted carbon nanotube yarn microelectrode; the scale bar is 10 µm. Adapted with permission from [82]. (**E**) Reduced-graphene oxide on top of a platinum electrode; the scale bar is 2 µm. Adapted with permission from [39]. (**F**) boron-doped diamond tip and parylene insulation; the scale bar is 100 µm. The scale bar of the inset picture is 10 µm. Adapted with permission from [54].

Carbon fiber is a graphitic carbon material with excellent mechanical properties and extraordinary electrical conductivity that has been extensively used in neurochemical sensing in the form of the CFM; see Figure 2B. Carbon fiber is extremely robust for its small size; it is able to remain straight without breaking upon implantation in the brain despite its subcellular dimension (approximately 7 µm in diameter). In addition, the rough surface of a single carbon fiber gives it a larger surface area for sensing compared to a similarly sized metal electrode. CFMs have been used for decades to detect DA in vivo with FSCV [57,69,70,71]. Additionally, various electrochemical techniques and coatings for CFMs have been developed to create CFM-based sensors to detect 5-HT, histamine, hydrogen peroxide, adenosine, melatonin, and many others [72,73,74,75,76,77,78,79].

Carbon nanotubes are the most widely explored carbon nanomaterial due to their unique mechanical and chemical properties, large surface areas, and extraordinary electrical conductivity. CNTs are rolled sp^2^ graphene sheets with nanometer diameters and micrometer lengths; see Figure 2A. The two types of CNTs, multi-wall (MW) and single-wall (SW) CNTs, can be synthesized by various methods, such as chemical vapor deposition, laser ablation, and arc discharge. Defects on the edge planes (tube ends) and basal planes (sidewalls) and the functional groups decorating these sites affect the electroactivity of CNTs significantly. In one study, a comparison was made between MWCNTs with and without edge-plane-like defect sites and different degrees of acid functionalization with cyclic voltammetry studies using potassium ferrocyanide (K_4_Fe(CN)_6_) and hexaammineruthenium (III) chloride (Ru(NH_3_)_6_Cl_3_) solutions respectively [80]. They found that edge-plane-like sites are main contributor of MWCNT electroactivity and that oxygen-containing functional groups inhibit the rate of electron transfer at these sites for the two standard redox probes. 

Additionally, the kinetics are believed to be faster from vertically aligned CNTs than randomly dispersed ones. For example, the oxidation and reduction peak separation for K_3_Fe(CN)_6_ was found to be 99 mV for randomly dispersed SWCNTs. For the same one-electron transfer reaction, a peak separation of 59 mV was reported when using a vertically aligned MWCNT modified electrode, indicating faster kinetics [81]. When acid-functionalized, CNTs can be used as a dopant in conductive polymer coatings to impart CNTs’ desirable sensitivity and fast kinetics to the electrodes. Taylor et al. coated PEDOT/CNT on CFM and used SWV for DA detection. The reported sensitivity of bare CFM was 0.21 ± 0.08 µAµM^−1^, whereas the PEDOT/CNT coating increased the sensitivity by more than 500 fold to 108 ± 9 µAµM^−1^. The LOD of PEDOT/CNT coated CFM was 2.03 ± 0.09 nM. The in vivo basal DA concentration in the rat striatum was reported to be 82 ± 6 nM [25]. Compared with the time-consuming and expensive CVD growth of CNT, this electrodeposition method provided a facile alternative and can be easily applied to any microelectrode array substrates by electropolymerization of PEDOT in the presence of CNTs. 

CNT yarn microelectrodes (CNTYM) are made by twisting and spinning CNT fibers together. CNTYM have been used as a new carbon nanomaterial for neurotransmitter detection as an alternative to CFM; see Figure 2D. A detailed electrochemical comparison between CNTYM and traditional CFM is available elsewhere [82]. CNTYM has a much rougher surface and is more sensitive to nearly all the analytes tested in their work. CNTYM also has an advantage over CFM in distinguishing electroactive species, as it exhibits sharper peaks in response to DA, AA, and other neurochemicals, due to faster electron transfer kinetics. Sensors incorporating CNT yarn have been recently reported for the detection of DA, 5-HT, and glucose [83,84,85]. 

Graphene, a single sheet of 2-dimensional carbon composed of sp^2^ carbon arranged in a hexagonal lattice, has extraordinary electrical, optical, and mechanical properties [86]. In a previous study, our group developed a PEDOT (poly(3,4-ethylene dioxythiophene)/graphene oxide (GO)-modified CFM for enhanced DA detection using FSCV in the dorsal striatum [87]. The PEDOT/GO layer was co-electrodeposited to the CFM surface with varying deposition times. With the increase of deposition time, the sensitivity to DA increased drastically and then plateaued. Modified electrodes exhibited a maximum of an 880% increase in sensitivity, and the LOD was improved from 218 ± 20 nM to 85 ± 9 nM. This enhancement of DA sensing performance comes from the increase in effective surface area and improvement in DA adsorption on GO. PEDOT/GO-coated electrodes also presented enhanced in vivo DA signals in response to electrical stimulation of the medial forebrain bundle.

Nanodiamond is a crystalline carbon nanoallotrope consisting of tetrahedral sp^3^ carbon atoms. Doped ND is a promising material in sensing due to its low charging current, wide potential window, good stability, and anti-fouling properties. Many substances have been detected using ND-based microelectrodes, including DA in vivo [40,46,88,89]. The Venton group optimized drop-cast ND coatings on a CFM and found that the carboxylated ND with a particle size of 15 nm gave the most sensitive DA response with FSCV detection. They showed that the ND coating reduced chemical fouling from 5-HT and 5-hydroxyindoleacetic acid and also decreased biofouling in tissue slices by 50% [90]. In addition, scientists at the Mayo Clinic reported the first use in humans of a polycrystalline boron doped diamond (BDD) microelectrode for recording in the thalamus; see Figure 2F [54]. The in vitro data showed distinct peaks for DA and adenosine using FSCV. After 6 days of in vitro use (corresponding to 5.2 million measurement cycles), the BDD electrode demonstrated a minimal decrease in sensitivity (−16%) while the CFM had completely lost sensitivity to DA. The resistance of nanodiamond to fouling makes it a highly promising electrode material for future long-term recordings in vivo.

#### 2.2.2. Metal Electrodes

Although carbon materials have many advantages, fabricating carbon-based electrodes or microelectrode arrays has been challenging because of the long, costly process of micromanufacturing and the difficulty in scaling up the fabrication of high-density carbon electrodes with 3D arrangements [94]. Metal electrodes do not offer the same advantages as carbon in terms of electrochemical stability and a wide electrochemical window, but they have some desirable chemical properties, such as the ease of adding surface modifications to immobilize biorecognition elements and electrocatalytic behavior towards specific redox active species [38,95,96,97]. In order to improve charge transfer rates, decrease oxidation potentials, and increase sensitivity while still being able to achieve good selectivity, metal electrodes modified with ionic liquids and novel polymers have attracted significant attention in the development of new electrochemical sensors [98,99,100]. As summarized in another review by [101], most of these works are limited to in vitro testing, showing enhancements in sensitivity, selectivity, and stability by utilizing novel modifications, but they lack in vivo validation. Some metals also have catalytic properties that can shift redox potentials into a more desirable range; the classic example of this is platinum’s catalytic effect on the reduction of oxygen in a Clark electrode, a technique that is still in use in clinical brain measurements today [102,103]. While oxygen’s standard reduction potential is quite high and highly dependent on pH, a Clark electrode [104] reduces oxygen at a platinum electrode at −0.6V, far away from potentials that may cause undesirable reactions [105]. The Clark electrode utilizes an oxygen-permeable membrane (cellophane in Clark’s original work, polyethylene more commonly today) to reduce biofouling. 

Additionally, platinum commonly serves as the substrate material for ROS or reactive nitrogen species (RNS) detection [106,107]. A PtIr/Nafion/oPD wire electrode for the acute recording of NO in Lewis lung carcinoma tumors in mice was reported [107], where a Nafion layer was dip coated and dried at 80 °C, followed by electrodeposition of oPD to enhance the selectivity against possible interferent molecules. They injected two types of NO donors to the tumor area. The decomposition of microinjected NO donors produced exogeneous NO molecules that were directly detected by the sensor using CA.

#### 2.2.3. Enzyme Biosensors

Enzyme-based sensors have been in use for decades [108]. Enzyme-based biosensors take advantage of the fact that enzymes have evolved with high specificity to their substrates; for example, previous studies suggested that glutamate oxidase (GluOx) is highly specific for Glu and the detected currents from 22 other amino acids are negligible [109,110]. The primary methods used for enzyme immobilization on a sensor’s surface are crosslinking, entrapment, and electrodeposition [111,112,113]. The most commonly used immobilization technique is crosslinking; this technique is relatively simple, and the resulting layer has strong adhesion to the sensor substrate. During immobilization, a small bead of solution is usually drop casted to the electrode’s surface or the electrode is dip coated in an enzyme solution, followed by a drying process. The electrodeposition method has attracted more attention due to the advantage of selectively coating specific sites on one MEA platform. Tian et al. electrodeposited both the mPD and gel-enzyme layers for recording Glu recording in the dorsal medullary nucleus of the solitary tract. The linear range of said sensor was 0.5–100 µM and the theoretical LOD was as low as 5 nM. The sensor successfully screened the amperometric responses from interferents such as AA, DA, UA, 5-HT, and catechol. [114]. Enzyme biosensors often take advantage of screening layers and self-referencing techniques to reduce the influences of interfering molecules [115,116,117], as detailed in Table 1.

**Figure 3:**
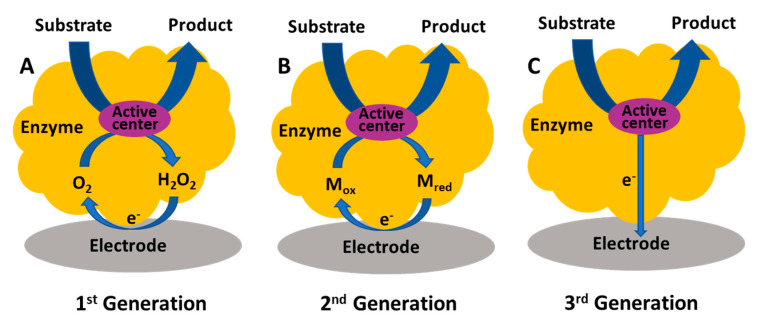
Representative schematics of different generations of amperometric enzyme biosensors. (**A**) First generation biosensor wherein the byproduct H2O2 is used for electron transfer. (**B**) Second generation biosensor wherein an artificial redox mediator is used for electron transfer. (**C**) Third generation biosensor wherein the enzyme itself is used for electron transfer.

Enzyme biosensors can be classified into three generations, and corresponding mechanisms have been well summarized in many review papers [118,119]. In brief, the first generation uses oxygen as the electron doner and detects the decreased oxygen or liberated H_2_O_2_; see Figure 3A. Similar oxidases include choline oxidase, pyruvate oxidase, lactate oxidase, and glutamate oxidase [120,121,122,123]. Second generation biosensors use a mediator (ferrocene derivatives or ferrocyanide, etc.) and thus are oxygen independent; see Figure 3B. Third generation biosensors rely on bio electrocatalysis, where direct electron communication between the enzyme redox center and the electrode occurs; see Figure 3C.

The first generation biosensor is the gold standard for enzyme sensors due to its facile procedure and good stability and sensitivity. In a recent study, ascorbic acid oxidase (AAOx) was used to consume the AA at a chronoamperometric sensor’s surface and detect the basal levels of AA (~200 µM) in the hippocampus by measuring the decrease in current [117]. New electrode materials utilizing catalytic properties to reduce analyte oxidation potentials have been developed for in vivo sensing. For example, a cobalt single-atom catalyst coated glassy carbon electrode was used to detect the oxidation of H_2_O_2_ from a glucose sensor at only +0.2 V, a −0.5 V shift from the standard oxidation potential used for amperometric detection of H_2_O_2_ [124]. Conducting polymers such as poly(2,5-di(furan-2-yl)thiazolo[5,4-*d*]thiazole)(PTTzFr) or electroactive Schiff based polymers with 2D near-single-atom thickness were also reported to help immobilize glucose oxidase (GOx) and detect the catalyzed oxygen reduction with good stability [125,126].

The second generation biosensors are less widely used in vivo because of poor stability of the mediator immobilization and possible toxic of mediator [127]; however, reports on the use of second generation biosensors have become common. A second generation, fully inkjet-printed disposable glucose sensor was recently constructed by integrating Nafion/ferrocene/GOx on top of poly(3,4-thylenedioxythiophene):polystyrene sulfonate (PEDOT:PSS). Glucose was detected linearly (28 µM–0.85 mM) through a cascade of electron transfer to PEDOT, which oxidized at +0.2 V vs. Ag/AgCl. However, the mediator and enzymes were suspected to leak out during continuous use and wash [128]. A similar study integrating a screen-printed glucose sensor on a polyethylene terephthalate (PET) substrate was also reported, wherein Prussian blue was used as the mediator. On-body glucose measurements in sweat were performed [129]. As second generation biosensors are currently limited by the stability of the immobilization of the mediator, second generation biosensors are rarely used in vivo. Future studies will aim to improve the stability of mediator immobilization.

Third generation biosensors are superior to the first two generations in that they are oxygen independent and mediator free; thus, they are also called direct electron transfer (DET) biosensors. The main difficulty of direct electron transfer from the redox center is the it is buried deep and insulated in the protein [130]. Bacterium-derived flavin adenine dinucleotide (FAD)-dependent glucose dehydrogenase (GDH) complex was frequently reported in recent decades as an option for the third generation biosensors. This FADGDH complex consists of an iron-sulfur subunit and heme units that would transfer electrons from glucose oxidation to the electrode’s surface [131]. A variety of FADGDH-based sensors, including amperometric [132], potentiometric [133], and impedimetric [134] sensors, have been reported. Employing direct electron transfer (DET) is considered the most ideal for use in electrochemical enzyme sensors [135]. The sensitivity of a third generation biosensor can be improved due to higher integration between the biomolecule and the electrode surface than in the previous two generations of sensors [136].

Multiple enzymes are sometimes employed in one biosensor array for either simultaneous detection of multiple neurochemicals or using cascades of reactions to detect one or more analytes [137]. For example, acetylcholine can be converted to choline by acetylcholinesterase (ACHE); choline oxidase (ChOx) then reacts with choline and produces H_2_O_2_ as a byproduct which can be detected [138,139,140,141]. A 4-channel microelectrode array for the detection of choline and acetylcholine in vivo in a rat striatum was reported by the Gerhart group, wherein all four sites of the platinum array were coated with mPD/ChOx and two of the sites were coated additionally with a layer of ACHE. Therefore, the choline signal can be obtained directly from Pt/mPD/ChOx and the acetylcholine signal was accessed by subtracting the signal of Pt/mPD/ChOx from that of Pt/mPD/ChOx/ACHE [120]. Similarly, recent in vivo studies in the rat’s frontal cortex, striatum, and hippocampus to detect the main inhibitory neurotransmitter GABA were reported by Arumugam’s team and Gerhart group. The difference between the signal from a Glu site (Pt/GluOx) and a GABA site (Pt/GluOx+GABase) was attributed to the H_2_O_2_ generated from GABA [142,143,144]. Technologies to simultaneously detect multiple neurochemicals by utilizing multiple enzyme MEA biosensors provide a platform to investigate complex neurochemical interplay dynamics in vivo.

#### 2.2.4. Aptamer Biosensors

**Figure 4:**
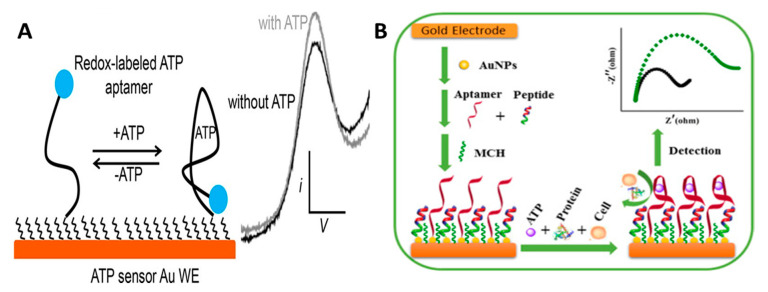
Schematic of an electrochemical aptamer-based biosensor demonstrating altered electron transfer efficiency upon binding of neurochemicals. (**A**) SWV detection of ATP with the aptamer fold and alter electron transfer of tag, thereby increasing the observed peak. Adapted with permission from [147]. (**B**) EIS detection of ATP with charge transfer resistance R_ct_ increased by ATP concentration. Adapted with permission from [148].

Aptamers are artificially synthesized nucleic acid sequences that can bind to a variety of molecules, including proteins, amino acids, drugs, and neurotransmitters. While most enzyme-based biosensors take advantage of the selectivity of enzymes that have evolved naturally, aptamers are created via artificial evolution. They are synthesized and isolated using an iterative process called systematic evolution of ligands by exponential enrichment (SELEX). A detailed explanation of the SELEX process for aptamer selection and a comparison between antibodies and aptamers can be found in a short review written by Ciara K. O’Sullivan [145]. Aptamers bind to target molecules by affinity binding, like antibodies; however, aptamers have advantages over antibodies in terms of high specificity, affinity, and stability, and once an aptamer has been developed, it can be repeatedly synthesized with high reproducibility. Additionally, because antibodies are typically created by injecting the target molecule into an animal to induce an immune response, small molecules that do not cause an antibody response and highly toxic compounds are better suited to aptamer-based sensor developments [146]. Electrochemical aptamer-based (E-AB) sensors are constructed by conjugating the ends of aptamers with functional groups that either enable aptamer surface immobilization or are electrochemically active for signal detection. An ATP E-AB sensor has been used for sensitive measurements (1–10 µM) in astrocyte cell cultures stimulated by Ca^2+^, glutamate, and ionomycin using SWV; see Figure 4A [147]. Luo’s group constructed a gold nanoparticle (AuNP) E-AB sensor by using a self-assembled ATP aptamer with an anti-fouling peptide and coated 6-mercaptohexanol (MCH) as a blocking agent to remove weakly bonded aptamers. They used charge transfer resistance R_ct_ determined by electrochemical impedance spectroscopy (EIS) as an index for quantifying the ATP concentration, where R_ct_ increased with ATP concentration; see Figure 4B. The reported linear range is 0.1 pM to 5 nM, and they attribute this low LOD to the AuNPs and the peptide that help retain the aptamer’s binding affinity [148]. An in vivo study of adenosine can be found in Zhang’s work, where they injected adenosine to the rat as a proof-of-concept experiment [149]. E-AB sensors were also developed for the detection of DA. Andrews’ group constructed flexible aptamer-field-effect-transistors for DA and 5-HT sensing with a 10 fM LOD [150]. However, most E-AB sensor work focuses on in vitro development and analysis only using serum or blood samples [151,152,153]. This is presumably due to the instability of E-AB sensors in vivo that results from the degradation of the recognition aptamer, and non-specific interferant adsorption on the sensor’s surface may cause significant drift [154]. Nevertheless, in vivo work describing the detection of cocaine or small drug molecules has been reported [95,155,156] and methods such as hydrogel coating [157], kinetic differential measurement [158], and dual-reporter approaches [159] have been explored to correct drifted signal.

### 2.3. In Vivo Challenges of Electrochemical Sensors

Electrochemical sensors have many advantages, including their relatively low cost, ease of manufacture, low detection limits, and high customizability. One of the main concerns with quantitative analyses in vivo using electrochemistry is the sensitivity. Electrode sensitivity may decrease due to either biofouling (e.g., protein adherence to electrode surfaces or fibrous or glia tissue encapsulation) or electrochemical fouling (e.g., irreversible electrochemical products adhere on the electrode surface or electrode material degradation due to electrochemical cycling) on the electrode surfaces. Electrode layers with antifouling or anti-interferent properties are often incorporated in electrochemical sensor design as an important component to protect surfaces from fouling. Chemical and physical approaches are the two main methods utilized in surface coating for an antifouling layer. Chemical approaches use highly hydrophilic systems to create a hydration layer that is resistant to nonspecific protein adsorption and reduces inflammatory responses. For example, hydrophilic leukocyte membranes and conducting polymers tailored with zwitterionic phosphorylcholine have been demonstrated to prevent biofouling in CFM in vivo [160,161]. Physical approaches rely on the engineering of filtration membranes or porous electrodes that exhibit size-related diffusion restrictions. To be more specific, polymers such as Nafion and polyvinylpyridine are charge exclusive coatings, and ortho, para, or meta poly-phenylenediamine (oPD, pPD, mPD), polypyrrole (ppy), polyaniline, cellulose acetate, and polyphenol work as size exclusive films [162,163,164,165]. Feng et al. developed a polytannic acid (PTA)-doped nanoporous conductive polyaniline (PANI) membrane-coated CFM for in vivo DA sensing in rat brains. The polymerized PTA-PANI layer is not only highly hydrophilic, but also cation-permeable. Thus, it alleviates non-specific adsorption of proteins while still allowing DA to reach the electrode [166]. Additionally, it was also suggested that engineering an electrode surface with pores of varying size would help tune the antifouling performance [167,168]. A comprehensive review discussing the recent advances in building antifouling sensors using chemical, physical, or biological antifouling strategies can be found elsewhere [169].

Since the brain is a complex environment where target analytes may coexist with many other interfering chemicals, selectivity is a great concern. Selective electrochemical sensors are an imperative requirement to guarantee quality recording and accurate quantitative analyses of chemical concentrations. In addition to membrane coatings that impede interferents, alternative methods that are frequently employed in in vivo studies include incorporating a catalyst that shifts the detection potential to a safe region or self-referencing techniques that subtract the background signal from the sensing site. However, many published in vivo electrochemical sensing works rely on the intrinsic ability of techniques such as SWV, DPV, and FSCV to provide distinguished peaks for multiple analytes, or else attribute the selectivity to the overwhelming presence of a single cell type that secretes the target analyte compared to other neurochemicals, and thus there is no need to utilize an additional selectivity strategy.

Another requirement in an in vivo electrochemistry study is to develop an electrode with minimal invasiveness and high spatial resolution to better understand cellular level neurochemistry and facilitate charge transfer at the electrode/tissue interface. Benefitting from micromanufacturing technology, various types of micro-scale electrodes have been developed for in vivo neurochemistry studies; however, the penetration of microelectrodes can still introduce some local tissue damage which may hinder the accuracy of neurochemical sensing. A micro-invasive probe (µIP) has been developed that markedly reduces inflammation markers and tissue damage. This micron-scale diameter CFM was used for stimulated DA detection for more than one year in vivo [170]. Furthermore, owing to the advancements in micro/nano-scale techniques, nanoelectrode fabrication has been made possible by utilizing methods including bottom-up manufacturing, electrochemical and flame etching of conical needles, deposition on nanopipettes, and laser-assisted nanowire pulling [171]. Nanoelectrodes not only reduce the mechanical damage, but also made it possible to perform single cell analysis. Single cell intracellular analysis of catecholamines and ROS/RNS has been achieved using bare or modified nanotip electrodes in living cells [172,173] and in vivo [174,175].

## 3. Optical Sensors

While electrochemical sensors are more common because of their low cost and ease of use, optical sensors also play important roles in neurochemical sensing due to their high temporal and spatial resolutions. Optical sensors frequently have reduced invasiveness or are completely non-invasive when compared to techniques requiring electrode implantation, making them particularly important for in vivo applications. The advances in optical microscopy and the growing numbers of fluorescent biomarkers and genetically encoded indicators have contributed to a detailed observation of cellular dynamics at extraordinary spatial and temporal resolution. Table 2 summarizes several optical in vivo studies along with the optical methods used, analyte, and LOD.

### 3.1. Fluorescence Sensors

Fluorescent dyes are chemical compounds that emit light upon absorbing light or other electromagnetic radiation energy. Traditional fluorescence sensors based on metal or organic dyes for catecholamine detection have been in use for decades [179,180]. However, several new categories of neurochemical sensors have been incorporated in this field. Carbon nanomaterials have stimulated much interest in optical electronics due to their extraordinary structures and physiochemical properties. SWCNTs wrapped with synthetic biomimetic polymers have been used for selective detection of DA in the near-infrared region utilizing corona phase molecular recognition, a mediated interaction between DA and SWCNT corona using the pinned polymer [181,182]. Chen et al. also reported a GO-based, photoinduced charge transfer, label-free near-infrared fluorescent biosensor for DA. Their detection mechanism relies on the significant quenching of near-IR fluorescence GO with a left shift attributed to attached DA [183]. Quantum dots (QDs) are nanometer-sized fluorescent crystals made from semiconductor materials that exhibit unique luminescent properties. Compared with traditional fluorescent reporters or organic dyes, QDs are reported to be 20 times brighter and exhibit size-dependent emission properties [184,185]. A nano biosensor for in vivo AA analysis based on nitrogen doped graphene quantum dots (NGQDs) and CoOOH nanosheets was reported recently [186]. The strong fluorescence emission at 445 nm displayed by NGQDs is suppressed by the CoOOH nano-quencher. AA can be detected by an increase in fluorescence intensity at 445 nm because AA effectively reduces CoOOH to Co^2+^, and thus removes the quenching effect. However, many QDs are highly toxic and not appropriate for in vivo studies. To address this problem, the development of simple and nontoxic methods for neurochemical sensing is highly desired. Conjugated polymer nano particles (CPNPs) are a more biocompatible option than QDs, with superior photostability. A CPNP for fluorescence sensing of DA in the ventricles of zebrafish larvae brains was developed [187]. When PBA (phenylboronic acid) on the CPNP surface is bound to DA, a photoinduced charge transfer occurs between the DA and the CPNP emissive core. This charge transfer leads to fluorescence quenching of the CPNPs. As a result, the fluorescence intensity decreases as the DA concentration increases, a “turn off” response. Other physiological sources of interference, such as AA, epinephrine, and NE, only trigger a negligible fluorescence response, confirming the DA specificity. When microinjected in the zebrafish larvae’s brain ventricles, CPNP showed a 96% decrease in fluorescence intensity in response to microinjected DA, 1 µM. Development of fluorescent reporters has significantly advanced in recent years, and fluorescence has become very common in sensor research. Fluorescent false neurotransmitters (FFNs) are optical reporters packaged together with target analytes into vesicles. FFNs contain moieties that can be recognized by the target neuron’s vesicular transport proteins. Thus, FFNs enable the visualization of neurotransmitters upon their co-release into the synaptic cleft following an action potential. FFNs have pushed forward the spatial resolution of fluorescent techniques down to the synaptic level, allowing single vesicle detection [188]. However, because FFNs do not provide direct neurotransmitter detection and provide limited information about the extracellular dynamics, FFNs are used typically in vitro in cell cultures or ex vivo in brain slices [189,190].

**Figure 5:**
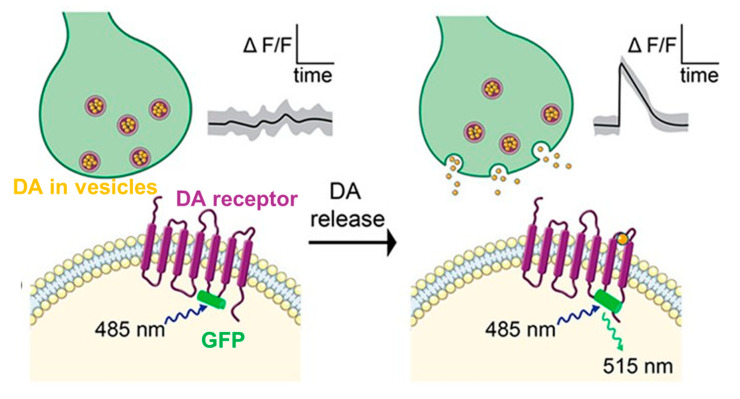
Schematic illustration showing the fluorescence increase of genetically encoded G protein coupled receptor-activation-based-DA sensor (GRAB_DA_) in response to extracellular DA. When DA is released from vesicles, it binds to the GRAB_DA_ receptor. The subsequent conformational change in the receptor alters the arrangement of the green fluorescent protein (GFP) associated with the receptor, resulting in a DA-dependent change in fluorescence. Reprinted with permission from [202].

Genetically encoded sensors rely on genetically encoded fluorescent proteins to quantify intracellular physiological events. The recent developments have enabled the measurement of pH, ion concentrations, and redox indicators or others [197]. Generally, genetically encoded sensors are used for the observation and measurement of neurochemical dynamics by monitoring the conformational changes of the expressed protein. As shown in Figure 5, in the G protein coupled receptor-activation-based-DA sensor (GRAB_DA_), a conformation-sensitive circular permutated green fluorescent protein (csEGFP), is coupled to a DA receptor. Upon DA binding, the receptor undergoes a conformational change which changes the arrangement of the EGFP and causes a fluorescent intensity change. GRAB_DA_ sensors have been reported to exhibit strong sub-second responses to extracellular DA with nM affinity and molecular specificity [195]. Furthermore, co-expression of GRAB and the calcium indicator GCaMP6 has enabled simultaneous measurement of DA release and Ca^2+^ activity in vivo [198]. Similar platforms have been developed for sensing Glu (fluorescent indicator protein for glutamate (FLIPE) [199] and intensity-based glutamate sensing fluorescent reporter (iGluSnFR)) [194], DA (dLight1) [200], NE (GPCR activation-based NE sensor, (GRAB_NE_)) [196], and Ach [193].

Modern multi-photon microscopy and miniaturized equipment allow the neurochemical dynamics to be monitored with these platforms in freely moving animals with minimal disruption to the tissue. Studies using genetically encoded sensor techniques reported successful in vivo imaging of Glu in rodent brains to study Glu transporters in Aβ plaques and Huntington’s disease models in freely moving animals at sub-second time resolutions [201]. With the advancement of fluorescent proteins and the utilization of miniature fast high-resolution microscopy, genetically encoded fluorescence sensors provide highly quantitative and highly selective imaging of neurochemistry in synaptic events and brain function in vivo.

In order to use genetically encoded sensors, cells of interest need to express these proteins. In vivo, this is generally accomplished by injecting a viral vector containing the desired fluorescent protein and an appropriate promoter into a genetically recombinant animal. This allows cell-type-specific imaging and single-cell-level sensing, a resolution that cannot be achieved by electrochemical methods. However, the narrow dynamic range and poor signal to noise ratio (<10%) can be concerns when using genetically encoded fluorescence sensors and are areas for future improvement [196].

### 3.2. Chemiluminescence Sensors

Chemiluminescence (CL) is the emission of light as a result of a chemical reaction. CL detection either uses a fluorescent compound that receives energy transferred from a reaction or creates a fluorescent product in its activated state, which in both cases will return to the ground state and release the excess energy as light.

CL has been utilized as a highly selective technique for chemical detection because of the high specificity of the reaction and the absence of autofluorescence interference caused by external light excitation. Recent advances in in vivo H_2_O_2_ imaging use peroxalate nanoparticles to chemically excite a reporter dye, which has low emission and is unstable in the presence of other ROS [192,203,204]. As an alternative, a semiconducting polymer nanoparticle (SPN)-based sensor was used for selective CL imaging of H_2_O_2_ in the mouse model of lipopolysaccharide (LPS)-induced neuroinflammation. The oxidation reaction between the peroxalate compound (TCPO) in the SPN core and H_2_O_2_ excites the SPs and ultimately leads to very strong luminescence [191]. Zhen et al. used a non-invasive in vivo spectrum imaging system for recording H_2_O_2_ in mice. When mixing polyfluorene-based SPN poly[(9,9′-dioctyl-2,7-divinylene-fluorenylene)-alt-] (PFPV) with various ROS in vitro, the near IR (NIR) CL response of PFPV from H_2_O_2_ was approximately 800-fold higher than those from OCl^−^, ·OH, ^1^O_2_, and ONOO^−^, indicating high specificity. A significant increase in CL intensity was measured after intracerebral injection of LPS in the striatum in vivo. The CL method does not rely on external light sources, and the NIR emission can be detected from outside of the body across the skull, both of which make non-invasive detection possible with a good limit of detection. In addition to the use in the quantification and localization of H_2_O_2_, online CL has also been coupled with techniques such as high performance liquid chromatography and microdialysis sampling for sensitive measurements of DA, NE, UA, and others using homogenized brain tissue or dialysate collected in vivo [205,206].

Electrochemiluminescence (ECL) is a type of chemiluminescence in which light emission is produced from energy transfer during electrochemical reactions. ECL reactions can be controlled by cycling the applied potential on the electrode because the reagents participating in the ECL reaction are produced by oxidation or reduction at a given potential. One study reported the use of dual-stabilizers-capped CdSe quantum dots (QDs) for accurate and sensitive determination of DA from samples in human urine and cerebral spinal fluid [207]. The degree of ECL intensity quenching responded to DA linearly from 10 nM to 3 µM with a LOD of 3 nM. In addition, the sensor did not respond to 10 µM of UA and AA interferants. They only noticed a 4.9% reduction in ECL intensity after 3 weeks’ storage in a refrigerator

### 3.3. In Vivo Challenges of Optical Sensors

Penetration depth of light in biological tissue is a common difficulty that limits the use of many optical biosensors because light can be absorbed, reflected, and refracted in brain tissue. This presents a challenge for detecting signals from deep brain structures [208]. Advances in implantable optical fiber probes and specialized lenses have made it possible to access deeper tissue for imaging, down to several mm from the surface of the brain [200,209,210,211]. Moreover, dyes, nanoparticles with luminescent properties, or viruses in the case of genetic encoders must be injected to the region of interest in animals, which is a time-consuming process, and the transfection efficiency is not always ideal. Unlike electrochemical probes, these genetic methods cannot be used directly in humans until proven safe in the future. Currently, the photostability of these genetic encoders is limited to several hours. The diversity of chemical signaling molecules adds additional challenges to their use in vivo. An ideal optical probe would exhibit a high affinity to the target analyte with a minimal signal from competing neurochemicals. Protein-based genetically encoded sensors are designed to target specific cell populations. However, the selectivities (ΔF/F_0_ of analyte to interferent) of these optical probes are usually 1–2 orders of magnitude less than those of electrochemical sensors, which is an area for further improvement. Furthermore, the relationship between fluorescence intensity and analyte concentration is often non-linear, making precise calibration of the analyte concentration difficult. Nevertheless, current developments in optical probes and novel assays are providing powerful, less-invasive tools for neurochemistry studies with high spatiotemporal resolution, and optical neuroscience research has enormous potential to grow as these challenges are addressed.

## 4. Alternative Methods

Microdialysis is a widely used technique for sampling fluid from brain tissue. A microdialysis probe consists of an inlet tube and an outlet tube connected by an implanted semipermeable membrane that is in the order of 100s of µm in diameter and several mm in length—up to several cm in human studies. In this technique, artificial cerebrospinal fluid is pumped into the inlet of the probe as a perfusate and passes into the semipermeable membrane region. Due to analyte concentration gradients across the membrane, extracellular analytes with molecule weights less than the cut-off value of the membrane are driven into the perfusate by diffusion. Membranes have a cutoff ranging from thousands to hundreds of thousands of Da that only molecules with a lower molecular weight can cross. In this way, larger potentially interfering molecules can be effectively filtered out of the neurochemical-containing perfusate (dialysate). Membrane materials vary based on target molecular weight and application, and include polyamide, cellulose, cuprophane, and polyethersulfone. After passing through the membrane region, the dialysate then flows out the outlet of the probe for external online (immediate) or offline (later) analysis. The most commonly used analysis tools are HPLC, capillary electrophoresis (CE), mass spectrometry (MS), and electrochemistry [212,213,214,215]. The combination of microdialysis with those techniques is a powerful tool for in vivo applications that allows the identification of multiple neurochemicals with high selectivity and ultra-low LOD [216].

An obvious disadvantage in using microdialysis is the time required for analyte sampling. Microdialysis typically leads to a total delay time of approximately 10–20 min [217]. Recent advances in system set-ups, capillary-scale HPLC columns, microfluidic systems, nL-level precision sample loop valves, and improvements in optimization have brought temporal resolution down to the sub-minute range [218,219,220,221,222,223] Therefore, microdialysis is a good choice for neuronal processes that last from minutes to hours, but not as good as electrochemical or optical sensors in capturing the fast dynamics of neurochemistry. Another issue regarding the use of microdialysis is accuracy of measurement. Since the technique depends on the diffusion through the membrane, the neurochemical concentration in dialysate is equal to the real extracellular concentration only when given enough time to stabilize, i.e., zero flow. The diffusion-controlled sampling mechanism explains why a lower concentration of analyte is always reported when compared to electrochemical methods [224]. The result is that microdialysis measurements are a tradeoff between temporal resolution and accuracy of measurement.

Like other implanted sensors, inflammatory microglia responses are initiated immediately upon the penetration of microdialysis probes, as revealed by two-photon live imaging. Immediately upon probe implantation, the blood brain barrier is ruptured, and local microglia enter a transition stage by extending their processes towards the implant and encapsulating the implant surfaces [30,225]. This acute response may lead to a cascade of inflammatory events, including the activation of microglia, the release of various molecules, and the formation of a glial barrier. As a result, the microdialysis probe will no longer be sampling from the normal brain environment and the glial barrier can hinder the analyte diffusion, both of which may confound the sensing results [226]. Significant reductions in electrically-evoked DA releases were recorded by a CFE attached to a microdialysis probe as soon as 2 h after probe implantation [227]. Recent advances in anti-inflammatory drug delivery through the perfusate [228,229,230] and ultrasmall microfabricated nano-probe development [219] have improved the tissue/probe interface, resulting in longer-lasting in vivo measurements. By perfusing the anti-inflammatory corticosteroid dexamethasone through the microdialysis probe, glucose levels in TBI model rats have been continuously monitored for upwards of 10 days without glial barrier formation [231].

Positron emission tomography (PET) is a minimally invasive imaging technique that uses radioactive isotopes to visualize chemical activity in tissue. After radioactive isotopes are injected into the body, they will specifically bind to targets. When the radioactive isotopes decay, the gamma rays produced by a positron and an electron annihilating each other are detected by a photomultiplier tube and can be used to infer the concentrations and locations of chemicals [232]. For example, carbon-11 (^11^C) and fluorine-18 (^18^F) have been widely used to label glucose, the main energy source for the human brain, and oxygen-15 (^15^O) can be used to label water molecules, which helps monitor blood flow [233].

PET is a highly sensitive method that measures radioisotope concentrations in the picomolar range. Studies have used [^11^C] raclopride to assess the DA changes because [^11^C] raclopride binds to the D_2_R receptor (a DA receptor) and uncouples from the receptor when DA is released. Previously, PET studies using [^11^C] raclopride as a radiotracer have revealed decreases in DA release in alcoholics in the ventral striatum and increased DA release in Parkinsonian patients with pathological gambling [234,235]. A detailed summary of PET studies on the use of receptor radioligands for assessing changes in DA, 5-HT, Glu, GABA, and Ach concentrations can be found elsewhere [236]. The disadvantages of this method are that PET has comparatively low spatial (a few millimeters) and temporal resolutions (tens of seconds to several minutes) when compared with other chemical measurement methods. Additionally, PET relies on the engineering of new radiotracers, and the typical half-life falls from minutes to hours.

Nuclear magnetic resonance (NMR) is a physical phenomenon in which nuclei in a constant magnetic field absorb and reemit electromagnetic radiation upon exposure to weak radio-frequency pulses. NMR has two applications, namely, magnetic resonance spectroscopy (MRS) and magnetic resonance imaging (MRI). As a non-invasive method, NMR has been used in adult human brains for determination of the approximate concentrations and locations of neurochemicals and their metabolites due to their unique chemical structures. It has been suggested that NMR is a volume-based method and measures neurochemicals at both intracellular and extracellular levels; thus, the analyte concentration reported using NMR usually falls in the millimolar range due to a dramatically higher intracellular concentration [214,237]. To study the fast dynamics of low concentration (nM–µM) neurochemical changes in disease models, NMR is not an ideal choice. However, it does provide useful information on brain metabolism and neurochemical distributions.

## 5. Designing an In Vivo Sensing Experiment

### 5.1. Comparing In Vivo Neurochemical Sensing Techniques

Each of the described neurochemical sensing techniques has its own application-specific advantages and disadvantages. In addition to the previously discussed parameters of limit of detection, temporal resolution, spatial resolution, and level of tissue damage, cost and other limitations may make a technique inappropriate for a specific application. The broad advantages and disadvantages of the techniques discussed in this review are summarized in Table 3. In vivo electrochemical sensing is extremely popular; electrochemical sensors are extremely customizable, and under the right conditions may be an inexpensive way to detect neurochemicals with fairly high temporal and spatial resolution and a low limit of detection. However, the pool of analytes available for detection is limited, and chronic recording is a challenge, as electrode performance may decay over time. Additionally, electrodes are inherently invasive and may cause tissue damage, though they can be manufactured to be exceedingly small.

As a potentially less invasive technique, an optical method may be a better option in a study where tissue damage is a critical concern. With comparable temporal resolution and limit of detection to electrochemical methods, and extremely high spatial resolution, optical methods are very attractive for studies probing small populations of cells or single cells. However, engineered biomarkers are required and a lack of chemical specificity can be an issue.

On the other hand, microdialysis is extremely flexible, as it can be coupled to any number of detection techniques. Microdialysis is a great option for analytes that are not electroactive and do not have any fluorescence reporter available, and it is particularly useful in studies in which multiple analytes must be detected simultaneously. However, microdialysis is inherently not quantitative and has very poor temporal resolution. Microdialysis also causes significant tissue damage, though, as discussed, there are strategies available to minimize this.

PET and NMR, in contrast, are both completely non-invasive, though they both suffer from poor spatial resolution and may be prohibitively expensive for many researchers to perform.

### 5.2. Addressing the Inflammatory Responses

As we have discussed throughout this review, the inflammatory response of the tissue to an implanted sensor is a critical concern. While some techniques, such as PET and NMR, are completely non-invasive, the bulk of neurochemical sensing studies involve an implanted probe, and the tissue response to the implantation may perturb the measurement, leading to inaccurate data or ultimately to a nonfunctional device. Several strategies for mitigating the inflammatory response involve either the local delivery of a drug to the area around the device, or the binding of an anti-inflammatory molecule to the device itself. Anti-inflammatory drugs such as dexamethasone [228,238], antioxidants such as melatonin [239] or superoxide dismutase mimics [240,241], and nitric oxide [242] have been employed as anti-inflammatory strategies for neural implants. A recent study applied therapeutic hypothermia after microelectrode implantation and significantly reduced the expression of inflammatory regulating cytokines and chemokines [243]. A further strategy to reduce inflammation is microfabricating ultrasmall sensors [244]. Avoiding blood-brain barrier damage has been shown to greatly reduce inflammation in rats. Since the average distance between blood vessels is approximately 50 µm, sensors are frequently constructed to be smaller than that critical dimension [245,246]. Soft and flexible sensors are also often employed to reduce mechanical mismatches between the sensor and the tissue [247,248,249]. However, sensors must remain mechanically robust enough to be able to penetrate brain tissue without bending or breaking. As a consequence, many highly flexible sensors require guide cannulas or stiff shuttles that may cause further damage [250].

## 6. Future Directions

Neurochemical sensors are a powerful toolbox of devices for neuroscience research in vivo. While some methods such as CFM detection of DA have been in use for decades, others are still in their relative infancy. As refinements to the sensors are continuously made, several trends are emerging. Modern microfabrication techniques are allowing researchers to create more channels on smaller platforms. Electrochemical recording at multiple brain sites using microfabricated array electrodes modified with carbon fiber, conducting polymers, or enzymes has been achieved [25,251,252]. This allows for multiplexed neurochemical monitoring while causing less tissue damage and thus less perturbation of the neurochemical being measured. A dual glucose/lactate microbiosensor for detecting metabolic changes during pathological cortical spreading depolarizations was developed and validated for in vivo use [253]. Cortical spreading depolarizations are dangerous events that can cause energy depletion in neurons in TBI patients. Lactate and glucose are both valuable targets to establish the severity of the TBI injury, as the glucose/lactate ratio is a marker for tissue metabolic stress and ischemia [254]. The multimodal microfabricated sensor was able to simultaneously detect a transient decrease in glucose and an increase in lactate during a spreading depolarization event. These types of multiplexed measurements on microscale probes are very attractive for monitoring the effects of multiple neurochemicals on behavior or disease and will likely remain a quickly advancing area of research. Multimodal measurements are also of interest to correlate electrical activity with chemical measurements. In a recent study, a microfabricated probe was able to penetrate to deep brain structures such as the non-human primate striatum for electrophysiology and neurochemical sensing, using Pt black electrode modifications for enhanced sensitivity to DA [255].

Additionally, new wireless data transmission systems are allowing animals to move untethered, potentially improving the quality of behavioral correlations with neurochemical measurements. Miniaturized electronics for wireless, untethered neurochemical sensing from untethered animals have been developed to detect oxygen and DA [23,256,257]. Wireless neurochemical sensing has also been employed in humans [258,259]. Space is often at a premium in human studies, making wireless data logging a very attractive option. Wireless data logging systems that meet the standard for hospital use are becoming smaller and cheaper. A recent battery-powered potentiostat and wireless data transmission system with integrated biosensors and a microfluidic system for use with microdialysis manufactured by the Boutelle group costs less than £500 to build [260]. The Tiny FSCV potentiostat and wireless transmission system costs less than $75 [261] and is made with commercially available parts.

One future goal of neurochemical sensing is to treat brain diseases via closed-loop neurochemical feedback [262,263]. For example, detection of DA levels during deep brain stimulation treatment for Parkinson’s disease may help tailor stimulation pulse waveforms to the specific patient based on the detected DA response for better control of basal DA levels [264], and relief of Parkinsonian symptoms with reduced side effects.

Other future efforts in neurochemical sensing may include (1) increasing the sensitivity and selectivity to ensure the measured signal is precisely from the analyte of interest, i.e., that you detected what you meant to detect; (2) developing more strategies for real-time monitoring of multiple analytes to better understand the dynamics and the interplay of chemicals in the local environment; (3) reducing invasiveness by utilizing novel soft or flexible materials and coating techniques that reduce inflammation while maintaining straightness for the implanted devices, including both electrodes and optical fibers or lenses for deep tissue imaging.

## Figures and Tables

**Table 1:** Electrochemical sensors for in vivo neurochemical measurement.

Electrode Material	Method	Analyte	LOD	Selectivity Strategy	Stability	In Vivo Study	References
GC/Co-SAC/Nafion	CA	Glucose	N/A	Exclusive layer + low potential	Acute use	Microdialysate from insulin injected rat striatum	[124]
PtIr/oPD/Chit-GluOx/AAOx/BSA	CA	Glu	44 nM	Exclusive layer + AAOx	7 days, 95% in fridge	Electrically evoked Glu in subthalamic nucleus of anesthetized rat	[116]
Au/Poly-DA/AuNP/MPA	CA	DA	50 nM	Exclusive layer	1 h, 100% in vitro	K+ evoked DA in striatum of rat	[38]
CFM/PTA-PANI	CA	DA	N/A	N/A	Acute use	Electrically evoked DA in medial forebrain bundle of rat	[166]
CFM/Nafion/oPD	CA	NO	6 nM	Distinct peaks	Acute use	NO release following NMDA stimulus in hippocampus of rat	[176]
CFM/CNT/Nafion	CA	AA	0.7 µM	Exclusive layer + low potential	Acute use	Basal AA in hippocampus of rat	[117]
Pt/GluOx-(GABase)/mPD	CA	GABA, Glu	0.2, 0.05 µM	Exclusive layer + self-referencing	Acute use	K^+^ and GABA transaminase inhibitor evoked GABA, Glu release in frontal cortex of rat	[143]
Pt/mPD/ChOx/(ACHE)	CA	Ach, Ch	0.18 µM	Exclusive layer + self-referencing	Acute use	K^+^ evoked Ch, Ach in striatum of rat	[120]
BDD	FSCV	Adenosine, DA	N/A	Distinct peaks	3 days, 93.3% in vitro	Mechanically evoked adenosine release in thalamus	[54]
CNTYM	FSCV	DA	13 ± 2 nM	Distinct peaks	4 h, 100%	Electrically evoked DA in caudate putamen of rat	[177]
CFM-PEDOT-PC	FSCV	DA	N/A	Distinct peaks	Acute use	Electrically evoked DA in medial forebrain bundle of rat	[161]
µIP-CFM	FSCV	DA	5.7 nM	Distinct peaks	>1 yr	Electrically evoked DA in medial forebrain bundle of rat	[170]
CFM/Nafion	FSCV	DA	N/A	Distinct peaks	2 h	Electrically evoked DA in striatum of rat	[24]
CFM	FSCV	H_2_O_2_, DA	N/A	Distinct peaks	Acute use	DA dynamics under modulation in striatum of rat	[53]
CFM/Chit	DPV	5-HT	1.6 nM	Exclusive layer + Distinct peaks	Acute use	Basal 5-HT in intestine of zebrafish	[178]
CFM/GR-FeTSPc	DPV	5-HT, DA	50, 20 nM	Distinct peaks	Acute use	Basal DA and 5-HT change in striatum of mouse	[45]
Tungsten/BDD	DPV	DA	50 nM	Distinct peaks	Acute use	Nomifensine induced DA in medial forebrain bundle of rat	[40]
CFM/PEDOT-CNT	SWV	DA	2.03 ± 0.09 nM	Exclusive layer + Distinct peaks	Acute use	Tonic and nomifensine induced DA in striatum of rat	[25]
Pt/rGO-AuNCs/adenosine aptamer/MB	SWV	Cocaine	NA	Aptamer sequence	3 h	Infused and IV injected cocaine in striatum of rat	[95]

Abbreviations: GC: glassy carbon; Co-SAC: Co single-atom catalyst; PtIr: platinum iridium; oPD: ortho-phenylenediamine; GluOx: glutamate oxidase; Chit: chitosan; AAOx: ascorbate oxidase; BSA: bovine serum albumin; Au NP: gold nanoparticle; MPA: 3-mercaptopropionic acid; PTA: polytannic acid; PANI: polyaniline; ChOx: choline oxidase; ACHE: acetylcholinesterase; BDD: boron-doped diamond; CNTYM: carbon nanotube yarn microelectrode; PEDOT: poly(3,4-ethylenedioxythiophene); PC: phosphorylcholine; µIP-CFM: micro-invasive probe of carbon fiber microelectrode; (GR-FeTSPc): graphene-iron-tetrasulfophthalocyanine; CNT: carbon nanotube; rGO: reduced graphene oxide; AuNCs: gold nanocluster; MB: methylene blue.

**Table 2:** Optical sensors for in vivo neurochemical measurements.

Sensor Material	Method	Analyte	LOD	Selectivity Strategy	Stability	In Vivo Study	References
Peroxalate TCPO+ PFPV + dye	CL	H_2_O_2_	5 nM	Affinity binding (chemical reaction)	Acute use	Intracerebral LPS induced neuroinflammation	[191]
Peroxyoxalate CPPO + dye	CL	H_2_O_2_	1 nM	Affinity binding (chemical reaction)	Acute use	LPS induced inflammation in ankle of tumor mice	[192]
GFP + Ach receptor	FL	Ach	100 nM	Affinity binding (conformational change)	4 h	Odor stim to *Drosophila* antenna lobe; Visual stim to mice cortex	[193]
NGQDs + CoOOH	FL	AA	1.85 µM	Affinity binding (redox reaction)	Acute use	Microdialysate from rat brain	[186]
*E. coli* GltI + GFP	FL	Glu	NA	Affinity binding (conformational change)	Acute use	Neuronal process in *C. elegans*. Behavior related transient Glu in mice	[194]
PFBT+PBA	FL	DA	38.8 nM	Affinity binding (boronic acid-diol recognition)	Acute use	Injected DA into zebrafish larvae brain ventricle	[187]
EGFP + DA receptor	FL	DA	NA	Affinity binding (conformational change)	Acute use	Odor and electrical stim to drosophila. Visual stim to zebrafish. Optogenetic, reward stim and sexual behavior triggered DA release in mice	[195]
EGFP + α-adrenergic receptors	FL	NE	NA	Affinity binding (conformational change)	Acute use	Looming-evoked NE release in the midbrain of live zebrafish. Optogenetically and behaviorally triggered NE release in the locus coeruleus and hypothalamus of mice	[196]

Abbreviations: FL: fluorescence; CL: chemiluminescence; PFPV: poly[(9,9′-dioctyl-2,7-divinylene-fluorenylene)-alt-]; TCPO: bis(2,4,6-trichlorophenyl) oxalate; CPPO: bis[3,4,6-trichloro-2-(pentyloxycarbonyl)phenyl] oxalate; NGDQs: nitrogen doped graphene quantum dots; PFBT: (poly[9,9-di(3′-aminopropyl)-2,7-fluorenyl-alt-4,7-(2,1,3-benzothiadiazole)]; PBA: phenylboronic acid; EGFP: enhanced green fluorescent protein.

**Table 3:** Comparison of different methods for in vivo neurochemical measurements.

Methods	LOD	Temporal Resolution	Spatial Resolution	Tissue Damage	Cost	Limitations
Electrochemical sensors	<1 µM	<1 s	<100 µm	Invasive	Inexpensive	Limited number of analytes. Performances decay with time
Optical Methods	<1 µM	<1 s	<1 µm	Less invasive	Inexpensive	Rely on engineering of biomarkers. Optical access needed
Microdialysis	<1 nM	<10 min	<1 mm	Invasive	Cheap	Flow rate affects accuracy. Fluidic setup needed
Positron Emission Tomography	<1 nM	<1 min	<1 cm	Non-invasive	Expensive	Rely on engineering of radiotracers. Short half-life time of tracers. Large equipment
Nuclear Magnetic Resonance	<1 mM	<1 min	<1 cm	Non-invasive	Expensive	Structurally similar compounds may be difficult to separate. Complex data analysis. Large equipment
